# Seafarers’ Quality of Life: Organizational Culture, Self-Efficacy, and Perceived Fatigue

**DOI:** 10.3390/ijerph15102150

**Published:** 2018-09-30

**Authors:** Jae-hee Kim, Soong-nang Jang

**Affiliations:** 1 Department of Nursing, Hyejeon College, 25 Daehak-gil, Hongseong-eup, Hongseong-gun, 32244 Chungcheongnam-do, Korea; jh6857@naver.com; 2 Red Cross College of Nursing, Chung-Ang University, 84 Heukseok-ro, Dongjak-gu, 06979 Seoul, Korea

**Keywords:** quality of work life, organizational culture, organizational support, self-efficacy, seafarers, culture-work-health model

## Abstract

Using the Culture-Work-Health model, this study investigates the factors influencing the quality of life of seafarers. This study conducted a survey of 320 seafarers who have lived and worked on a ship for more than six months. This self-administered questionnaire included questions on organizational culture and support, self-efficacy, perceived fatigue, as well as the quality of work life. Organizational culture and self-efficacy were identified as factors affecting the quality of work life, while organizational support was found to have an indirect effect through self-efficacy and perceived fatigue. The final model accounts for 63.1% of the variance in seafarers’ quality of life. As such, this study shows that self-efficacy is important for the quality of life of seafarers, having both direct and indirect effects. Moreover, organizational support may prove to be the primary intervention point for relieving perceived fatigue and enhancing self-efficacy, thus improving the quality of work life.

## 1. Introduction

An individual’s quality of life is directly influenced by their job, which shapes both their economic and health status. Indeed, job-related stress is known to have a negative influence on the health and quality of work life [1]. Seafarers can spend more than six months onboard a ship once leaving port. They are typically exposed to a poor working environment—with high noise levels due to the ship’s onboard operations—while having to cope with physiological changes resulting from a three-shift work schedule. Meanwhile, the diverse and rapid changes to the natural environment while at sea make it difficult to maintain physical homeostasis [2]. Seafarers thus endure a highly stressful work environment and a significant degree of fatigue relative to other areas of employment. The accumulated stress and fatigue have a direct negative effect on seafarers’ health that may threaten both their own safety and that of their colleagues, and lead to operational accidents. 

These issues are compounded by the current conditions in the shipping industry. Onboard cultural clashes occur frequently, because of the multinational workforce. Moreover, as a result of the reinforcement of the International Convention and the depression in the shipping economy, there is a lack of skilled workers, which only exacerbates job-related stress and fatigue among seafarers responsible for onboard operations, negatively impacting physical and mental health [3,4]. In many cases, seafarers find themselves unable to alleviate their stress through positive means, often resorting to alcohol or cigarettes, and sometimes leading to addiction of these substances [5]. As a result of this particular working environment, seafarers tend to feel more deprived relative to those in other occupations [6].

Enhancing seafarers’ support system for a better working environment may result in higher subjective satisfaction with their workplace [7], which would lead to greater organizational harmony [8]. Considering the unique nature of maritime occupations, in which seafarers are required to operate efficiently in the ship’s socially-isolated environment and successfully perform tasks to increase subjective satisfaction, it can be argued that maintaining self-efficacy is essential [9]. Increasing internal job satisfaction and positive self-management by raising seafarers’ self-efficacy will enable long-term efficiency in organizing and managing the maritime industry. That is to say, the benefits are not limited to improving an individual’s ability to cope with stress and fatigue while raising their subjective satisfaction; they also positively impact the industry as a whole [9]. 

Organizational culture needs to be understood from the perspective of organizational and individual health, as well as organizational policy—that is, the particular set of values and beliefs determining an organization’s behavioral objectives and methods used to manage employees [10]. Indeed, organizational culture is a very important issue for employees of a maritime company. However, there is a marked lack of specific research on seafarers’ attitudes and behaviors, and their effects on the organization [11]. In effective human resource management, support is crucial for increasing the health and quality of seafarers’ work-related life. Accordingly, what can be confirmed as a variable in the organizational culture influencing the quality of a seafarer’s work life, as well as health in a specific working environment, can be seen as providing valuable basic data to construct an effective intervention. 

This study uses the Culture-Work-Health model (CWHM) developed by Peterson and Wilson [10] to identify these variables of the maritime organizational culture, thereby showing how organizational culture and health impact the work life quality of seafarers. The core concept of the CWHM concerns the organizational culture, structure, and function of the management system, organizational health, worker health, and quality of work life. The factors that determine the health of an organization and individual are found in the cultural elements of the organization. Workplace unit culture, job stress, personal and workplace health, and happiness form the conceptual bases of the model. Unlike Demand-Control theory, which has traditionally emphasized factors that determine job stress levels, the CWHM focuses on human relationships, and satisfaction with bosses and/or colleagues who affect the health of workers, and it also explains the association between culture, work, and health [10]. 

By implementing and verifying this model, this study seeks to provide basic data regarding human resource management to improve seafarers’ quality of work life. Moreover, this study reveals and discusses the influential factors in organizational culture and support, and how self-efficacy and health impact the quality of work life in the maritime industry. In ship organization, there is a rigid job delivery and transfer system in which job delegation is based on the hierarchy of captain and seniors [12]. Therefore, the influence of senior-level communications, such as those of captains and chief engineers about job-related attitudes of the other ship members, is greater than in other organizations [12,13,14]. In an industry that is highly dependent on human resources, organizational culture and support are important to improve the health and quality of life of core workers. Identifying variables affecting the quality of work life associated with maritime organizational culture will have important implications for providing health interventions to workers in occupational settings similar to those found in the maritime industry. 

## 2. Methods

### 2.1. Study Design and Data Collection

**Figure 1:**
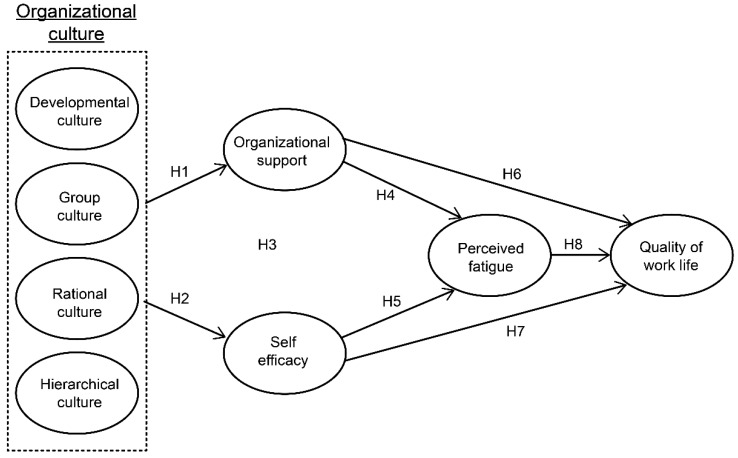
A conceptual framework based on Peterson and Wilson’s Culture-Work-Health model (CWHM).

Figure 1 presented the conceptual framework of this study by utilizing a hypothetical model based on the CWHM [10]. Using a self-administered questionnaire, this study surveyed seafarers who have worked onboard a ship for more than six months and belong to a shipping firm. Inclusion criteria is all seafarers who can read and respond to the questionnaire. Exclusion criteria is those who have boarding experience less than six months or who do not agree with the survey response. We purposely used volunteer-based sampling methods. The researcher conducted the survey with the seafarers, who were gathered for continuing education under the permission of the head of the shipping company education department. The researcher directly explained the purpose and significance of the research to minimize errors that could occur during the survey process. The study was explained to the participants, who were made aware that they could reject or stop participation at any point without repercussion. Approximately 320 questionnaires were disseminated, with 40 excluded because of incomplete answers; this resulted in a total of 280 questionnaires accepted for empirical analysis. The survey was conducted over a period of two months from 30 June 2016 to 31 August 2016, following the approval of the Chung-Ang University Research Ethics Committee (IRB No: 1041078-201605-HR-095-01).

### 2.2. Measurements

CWHM is a conceptual framework based on the assumption that quality of work life is determined by organizational culture, management system structure and behavior, and health. The predisposing variables of the management system are organizational culture, and the results of the management system are perceived fatigue and quality of work life. Organizational support and self-efficacy are mediators between organizational culture and perceived fatigue and quality of work life. Therefore, variables for the model used in this study, which affect quality of work life, are: perceived organizational culture, organizational support, seafarers’ self-efficacy, and perceived fatigue (see supplementary material).

#### 2.2.1. Organizational Culture

The organizational culture tool was modified and extended by Song on the basis of the organizational culture model developed by Quinn and McGrath [15,16]. In this study, organizational culture was assessed on a 5-point Likert scale and was composed of 17 items in four categories: developmental (five questions), group (five questions), rational (three questions), and hierarchical (four questions) organization. With Cronbach’s α = 0.959, the sub-factors are: developmental, 0.901; group, 0.906; rational, 0.855; and hierarchical, 0.849.

#### 2.2.2. Organizational Support

We used McMillan’s concept of gauging organizational support, first adapted by Loi et al. and modified by Nam [17,18]. This tool was based on a 5-point Likert scale and consisted of ten items related to emotional support and instrumental support. With Cronbach’s α = 0.942, the sub-factors are: emotional support, 0.867; and instrumental support, 0.919. 

#### 2.2.3. Self-Efficacy

This study used the self-efficacy measurement translated by Cho et al. and effectively used by Chen, Gully, and Eden [19]. Self-efficacy comprised a total of nine items based on a 5-point Likert scale, with a higher score equating to higher self-efficacy. In this study, Cronbach’s α = 0.914.

#### 2.2.4. Perceived Fatigue

This study measured the perceived fatigue of seafarers using the short form of the tool developed to measure an operator’s perceived fatigue, which was developed by Park et al. [20]. It was comprised of five items—three questions on mental fatigue and two questions on chronic fatigue—measured on a 5-point Likert scale, wherein a higher score indicated higher perceived fatigue. The reliability of the perceived fatigue in this study is Cronbach’s α = 0.853. The sub-variables showed mental fatigue at 0.730, and chronic fatigue at 0.777.

#### 2.2.5. Quality of Work Life

This study utilized a modified version of the Psychological General Well-Being Index (PGWBI-S), revised by Grossi et al. and suitable for seafarers [21,22]. This Index is composed of six items measured on a 5-point Likert scale, wherein a higher score indicates a higher quality of work life. In this study, Cronbach’s α = 0.893.

#### 2.2.6. General Characteristics

General characteristics included age (grouped into 20–30s, 40s and over), gender, and education (grouped by below junior college graduate level, university graduated or over). Work related factors included working years and job position. Other health behaviors included smoking status and stress level. We measured stress level by four responses; very little, little, high, very high. We further categorized these responses as either high (high and very high), or little (very little and little). 

### 2.3. Hypotheses

Based on both the literature and measurements identified above, we formulated eight hypotheses:

**Hypotheses** **1 (H1).** The organizational culture of seafarers will have a positive effect on organizational support.

**Hypotheses** **2 (H2).** The organizational culture of seafarers will have a positive effect on self-efficacy.

**Hypotheses** **3 (H3).** Organizational support will have a positive effect on the self-efficacy of seafarers.

**Hypotheses** **4 (H4).** Organizational support will a have negative effect on the perceived fatigue of seafarers.

**Hypotheses** **5 (H5).** Seafarers’ self-efficacy will have a negative effect on perceived fatigue.

**Hypotheses** **6 (H6).** Organizational support will have a positive effect on seafarers’ quality of work life.

**Hypotheses** **7 (H7).** Seafarers’ self-efficacy will have a positive effect on the quality of work life.

**Hypotheses 8** **(H8).** Seafarers’ perceived fatigue will have a negative effect on the quality of work life.

### 2.4. Statistical Analysis 

An analysis of the seafarers’ general characteristics and descriptive statistics was performed using frequency, percentage, mean, and standard deviation. The factors affecting seafarers’ quality of work life were analyzed on the basis of CWHM and utilized a structural equation model to verify the hypotheses. The internal consistency of each variable was verified via Cronbach’s α. We also tested the validity—including construct validity—of each variable forming the model, as well as the construct reliability and discriminant validity according to confirmatory factor analysis. The analysis was performed using SPSS 20.0 (SPSS Inc., Chicago, IL, USA) for Windows and AMOS 20.0 (IBM SPSS, Chicago, IL, USA). 

## 3. Results

### 3.1. General Characteristics of the Seafarers

The general characteristics of the 280 respondents are provided in Table 1 below. The majority of respondents (225 or 80.4%) were in their 20s and 30s. In terms of educational level, 163 (58.2%) of the respondents had received a college education or below. With regard to employment, regular full-time workers numbered 192 (68.6%); while in terms of work experience, 215 (76.8%) of the respondents had worked for ten years or less. With regard to position, second and third officers/engineers numbered 252 (90.0%). The majority of respondents were smokers, with 217 (77.5%) smokers and 63 (22.5%) non-smokers. A majority of the respondents (235 or 82.5%) indicated that they were under significant stress.

### 3.2. Confirmatory Factor Analysis for the Verification of Structural Model

A confirmatory factor analysis was performed to establish the validity of each measurement tool. The parameter estimation method utilized maximum likelihood. To evaluate the goodness of fit in the structural model, we used the following representative indexes, which show the explanatory power of the model: Goodness of Fit Index (GFI), Adjusted Goodness of Fit Index (AGFI), Normed Fit Index (NFI), Incremental Fit Index (IFI), Tucker-Lewis Index (TLI), Comparative Fit Index (CFI), Root Mean Square Residual (RMR), and Root Mean Square Error of Approximation (RMSEA). A goodness of fit of more than 0.9 tends to be acceptable in GFI, AGFI, NFI, IFI, TLI, CFI. For RMR, one less than 0.5 is an acceptable value, and for RMSEA, less than 0.08 is acceptable. Given that the goodness of fit is less than 0.05 in this study, the model is recognized as having a good fit.

### 3.3. Analysis of the Measurement Model

When the model’s factor loading was measured, the β-values were all above 0.4, indicating that the composition of the items was valid. To test convergent validity, we measured the values for construct reliability (CR) and average variance extracted (AVE). The convergent validity of a model can be proven when the CR and AVE values are above 0.7 and 0.5, respectively. The CR and AVE values of the model satisfied the conditions for convergent validity, thus proving the model’s convergent validity. Pearson’s *r* was < 0.90 for organizational culture, < 0.939 for organizational support, < 0.954 for self-efficacy, < 0.921 for perceived fatigue, and < 0.938 for quality of work life. Thus, the conditions for discriminant validity were satisfied, and the discriminant validity of the model was proven.

### 3.4. Parameter Estimation Results of the Structural Model 

**Figure 2:**
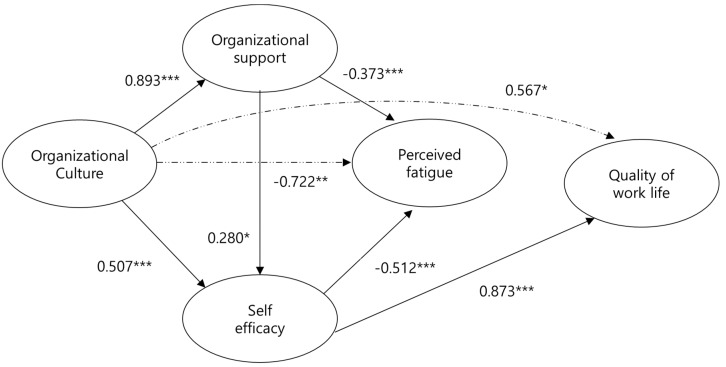
The relationship between organizational culture, organizational support, self-efficacy, perceived fatigue, and quality of work life among seafarers based on Peterson and Wilson’s Culture-Work-Health model (CWHM). Note: *p < 0.05, **p < 0.01, ***p < 0.001. Solid line: Direct effect, Dashed and Dotted Line: Indirect effect

In analyzing the goodness of fit, the initial model was found to satisfy all fitness standards; the estimation results of the parameter value in the structural model are provided in Table 2 and Figure 2. 

H1: The hypothesis that the organizational culture of the seafarers will have a positive effect on organizational support was statistically significant (t = 17,031, *p* < 0.001) and the hypothesis was supported (β = 0.893). 

H2: The hypothesis that the organizational culture of seafarers will have a positive effect on self-efficacy was statistically significant (t = 3.621, *p* < 0.001) with the direct effect (β = 0.507).

H3: The hypothesis that organizational support will have a positive effect on the self-efficacy of the seafarers was statistically significant (t = 1.986, *p* < 0.047). The direct effect was supported by β = 0.281. 

H4: The hypothesis that organizational support will have a negative effect on the perceived fatigue of seafarers was statistically significant (t = −4.484, *p* < 0.001) with β = −0.373.

H5: The hypothesis that self-efficacy of seafarers will have a negative effect on perceived fatigue was statistically significant (t = −5.80, *p* < 0.001) with β = −0.512.

H6: The hypothesis that organizational support will have a positive effect on the work-related quality of life of the seafarers was not statistically significant (t = −0.811, *p* < 0.417).

H7: The hypothesis that the self-efficacy of the seafarers will have a positive effect on work-related quality of life was statistically significant (t = 5.94, *p* < 0.001) with β = 0.873.

H8: The hypothesis that the perceived fatigue of seafarers will have a negative effect on work-related quality of life was not statistically significant (t = 0.132, *p* < 0.895).

In summary, of the eight hypotheses established in the hypothetical model of this study, six were supported (Table 2, Figure 2). As a direct consequence of estimating the parameter value, it was shown that the value of β is 0.893 (*p* < 0.001) in the influence of organizational culture on organizational support, β is 0.507 (*p* < 0.001) in the influence of organizational culture on self-efficacy, β is 0.281 (*p* < 0.05) in the influence of organizational support on self-efficacy, and β is 0.873 (*p* < 0.001) in the influence of self-efficacy on the quality of work life. This shows a significant positive influence. Moreover, it was indicated that β is −0.512 (*p* < 0.001) in the influence of self-efficacy upon perceived fatigue, and that β is −0.373 (*p* < 0.001) in the influence of organizational support on perceived fatigue. This shows a significant negative influence. 

In terms of indirect effect, β is 0.567 (*p* < 0.05) in the influence of organizational culture on the quality of work life, indicating a significant positive influence. The value of β in the influence of organizational culture on perceived fatigue was shown to be −0.722 (*p* < 0.05), inferring a significant negative influence. The judgment of significance in a direct effect was implemented via a bootstrapping test. 

## 4. Discussion 

This study implemented a comprehensive structural model by confirming the antecedent factors in the quality of work life of seafarers based on the Culture-Work-Health model, and identified the relationship between these variables. Organizational culture had a significant positive negative effect on perceived fatigue (β = −0.722, *p* < 0.05) through organizational support and self-efficacy, (*p* < 0.05), which was significantly positive for indirect effect. Organizational support was explained by organizational culture (79.8%), and self-efficacy was explained by organizational culture and organizational support (59.1%). Perceived fatigue was explained by organizational culture, organizational support, and self-efficacy by 68.2%. Quality of work life is explained by organizational culture, organizational support, self-efficacy, and perceived fatigue by 63.1%. The findings and suggestions of this study can be summarized in three main points:

First, organizational culture has a significant positive influence upon organizational support, which corresponds to the management system structure of a shipping enterprise. Organizational support was shown to have an explanatory power of 79.8% through the components of organizational culture. Self-efficacy, which corresponds to behaviors, positive influence received from organizational culture, and organizational support, was shown to have an explanatory power of 59.1% through the components of organizational culture and support. This confirmed that seafarers’ organizational culture is a significant factor influencing the perception of organizational support onboard a ship. Moreover, while the seafarers have an environmental element available for social exchange, they simultaneously identified that organizational culture and support can positively boost their self-efficacy. Organizational culture is a factor influencing organizational support [23]. Organizational members’ innovation behavior [14], strengthening group coherence, [11] might be one of the mediators in this relationship. In this study, we could not confirm the other directions between organizational culture and self-efficacy based on the CWHM, as Krajcsák (2018) concluded that self-efficacy and organizational support also transform the organizational culture [24].

Second, seafarers’ self-efficacy, which corresponds to the behaviors of the maritime industry’s management systems, was found to have a negative impact on perceived fatigue, indicative of the health of seafarers, but found to have a positive effect on their quality of work life. The negative effect on perceived fatigue implies that higher self-efficacy in seafarers leads to lower perceived fatigue and higher job enjoyment. It seems likely that maritime enterprises are in need of support and planning to improve the self-efficacy of their employees. An investigation of occupational health among nurses, for instance, found that job-related stress is associated with workplace culture and may lead to presenteeism [25]. Similarly, an examination of the relationship between self-efficacy and stress, quality of life among workers in charge of signaling lends credibility to the study’s findings [26]. Moreover, a study that used the CWHM to evaluate nurses who worked in three shifts, like the seafarers of this study, similarly found that organizational culture affected health [27]. Maritime industry executives need to strengthen appropriate organizational support by increasing encouragement and compensation in the organizational dimension to elevate the self-efficacy of seafarers, as well as through cementing and enhancing the organizational support perceived by seafarers. Further research is needed to construct a viable plan for positively reinforcing the management systems in terms of the developmental, rational, hierarchical, and group categories—that is, the sub-elements of the seafarers’ organizational culture [13].

Third, organizational support, which corresponds to the structure of the maritime industry’s management systems, was found to have a significant negative effect on perceived fatigue, indicating the health of the seafarers. This implies that organizational support and encouragement for seafarers’ job satisfaction provided by the maritime industry executives ultimately impacted their health. As such, improving organizational support will effectively enhance the efficiency of the maritime organization, which is highly dependent on human resources. Accordingly, a maritime enterprise should have great interest in the health and welfare of its employees [28].

Self-efficacy was shown to have a positive effect on the quality of work life, while organizational support and perceived fatigue were indicated to have a statistically insignificant effect on the quality of work life. This implies that a welfare service or corporate-level support perceived by seafarers is a factor that positively impacts self-efficacy and health, while having an indirect effect on the quality of work life. However, there are several studies whose findings contrast with the findings of this study. An investigation of shipyard workers, for instance, found that fatigue impacted psychological wellbeing [29], while another study examining production workers who performed shift work found that fatigue affected the quality of work life [30]. In contrast, this study found that perceived fatigue had no direct impact on quality of work life. Arguably, this is attributable to “the influence of healthy workers,” because only seafarers in good physical and mental health are allowed onboard due to the rigors of this occupation [31]. This may be “an impact of a healthy worker” because only the subjects in who are physically and mentally healthy can work on the ships. Moreover, health can be a means of life for seafarers. Consequently, there is a need to use a health-based approach, which considers the seafarers’ characteristics. It is a condition to board the ship that a seafarer should be physically healthy. Moreover, it is difficult to maintain a healthy physical condition for the six-month period onboard the ship, which is characterized by difficult working conditions and the possibility for many health situations to arise. Accordingly, the maritime industry must seek a solution that takes into consideration the problem of managing and maintaining the health of seafarers. The health issues facing the group or society to which seafarers belong must be contemplated from a different viewpoint. Hence, to promote productivity in maritime enterprises by improving seafarers’ quality of work life, it is necessary to construct a plan for a welfare dimension that systematically promotes the health of seafarers.

There are several limitations to this study. Notably, there is still insufficient data regarding the variable measured to fully explain seafarers’ quality of work life. Thus, verification through further repeated research is needed. In addition, exactly what perceived fatigue indicates for seafarers’ health may be problematic. Verification through diverse and repetitive research is required on a scale adequate to represent the health of seafarers. There is also a need to pay close attention to the representativeness of a scale, which measures the structures and behaviors in maritime management systems. This requires further research confirming scale verification. Furthermore, this study used non-probability convenience sampling among maritime employees of a shipping firm in South Korea. Therefore, this study did consider differences, such as corporate scale and regional characteristics. Thus, there is a limitation in generalizing the results of this study to all Korean seafarers. Moreover, this study verified its model by collecting materials cross-sectionally, and is thus limited in its ability to explain the relationship between relevant variables. Hence, close attention must be paid to the interpretation. In our analysis, organizational culture is the most important influence on quality of work life. Korean seafarers showed strong task-oriented culture; therefore, ways to reduce job stress should be investigated under their specific organizational culture.

## 5. Conclusions 

This study investigated an influential factor in the quality of work life experienced by seafarers based on the Culture-Work-Health model. Organizational support may be the first intervention point in relieving perceived fatigue and enhancing self-efficacy, and ultimately inducing a positive impact on the quality of work life. For workers in a specific environment, such as those who work for long periods onboard a ship, the development of a health management intervention program at their workplace is necessary. An education and training program for the promotion of health is necessary for all seafarers, as is assigning health coordinators to vessels that perform long-haul shipping. Most importantly, organizational support for these health promotion programs should be made a top priority to ensure the mental and physical health of seafarers. 

## Figures and Tables

**Table 1:** General Characteristics.

Classification		N (=280)	%
Age	20s–30s	225	80.4
	40s and over	55	19.6
Academic background	Below junior college graduate level	163	58.2
	University graduate	117	41.8
Employment type	Full-time position	192	68.6
	Contract worker	88	31.4
Working years	Less than 10 years	215	76.8
	More than 10 years	65	23.2
Job position	2nd and 3rd officer/engineer	252	90.0
	Senior officers (captain, chief officer & engineer, first engineer)	28	10.0
Smoking	Yes	217	77.5
	No	63	22.5
Stress	Less	45	16.1
	A lot of	235	83.9

**Table 2:** Parameter Estimation Results of the Structural Model.

						D	I	Total	SMC	D	I
		B	S.E.	C.R.	*P*	β	β	β		Hypo.	
Organizational support	Organizational culture	0.900	0.053	17.031	*p* < 0.001	0.893		0.893 ***	0.798	A	
Self-efficacy		0.392	0.108	3.621	*p* < 0.001	0.507	0.251	0.758 *	0.591	A	R
Perceived fatigue							−0.722 *	−0.722 *	0.682		A
Quality of work life							0.567 *	0.567 *	0.631		A
Self-efficacy	Organizational support	0.215	0.109	1.986	0.047 *	0.281		0.281		A	
Perceived fatigue		−0.318	0.071	−4.484	*p* < 0.001	−0.373	−0.144	−0.517 *		A	R
Quality of work life		−0.070	0.086	−0.811	0.417	−0.092	0.236	0.144		R	R
Perceived fatigue	Self-efficacy	−0.569	0.098	−5.8	*p* < 0.001	−0.512		−0.512 ***		A	
Quality of work life		0.870	0.147	5.940	*p* < 0.001	0.873	−0.009	0.864 *		A	R
Quality of work life	Perceived fatigue	0.016	0.123	0.132	0.895	0.018		0.018		R	

Note: *p < 0.05, ***p < 0.001. SMC: squared multiple correlation; D: direct; I: indirect; R: rejection; A: adoption.

## Supplementary Materials

The following are available online at http://www.mdpi.com/1660-4601/15/10/2150/s1, Survey paper.
