# Supplementary material for: Seafarers’ Quality of Life: Organizational Culture, Self-Efficacy, and Perceived Fatigue
Source: Int J Environ Res Public Health. 2018 Sep 30;15(10):2150. doi: 10.3390/ijerph15102150 (PMC6210239; doi:10.3390/ijerph15102150)
Supplement: Supplementary file 1 [file ijerph-15-02150-s001.pdf]

survey paper

The Informed Consent Form

The Purpose and Content of the Study

This questionnaire used in the study is to analyze what factors affect the quality of life of marine officers in the marine industry.

You are assured that everything of your completed questionnaire will be kept anonymous and confidential under Articles 33 and 34 of the Statistics Act, and that it will be used for academic research purposes only. There is no right or wrong answer to the questions, and it will be highly appreciated if you take time to give your opinions on every question.

Thank you so much for your valuable time.

I've fully explained about this study and understand it, and I've given sufficient answers to every question I asked. I agree to participate in this study voluntarily after due consideration.

Date:

Signature:

◈ If you have any questions, please feel free to contact the principal investigator mentioned below any time.

◈ If you decide to participate in this study and have any questions on your rights, please contact the principal investigator mentioned below.

H.P: 010-2731-4886

Ⅰ. The followings are about organizational culture. Please mark your answer with a V shape.

organizational culture	Very Poor	Poor	Fair	Good	Very Good
1.The shipping organization values innovative thinking and ideas.	①	②	③	④	⑤
2. The shipping organization takes the risk of a new attempt.	①	②	③	④	⑤
3. The shipping organization places importance on creative and adventurous attitude.	①	②	③	④	⑤
4. The shipping organization accepts new ideas when work is done.	①	②	③	④	⑤
5. The shipping organization promptly copes with the changes of work environments and coordinates them.	①	②	③	④	⑤
6. The shipping organization keeps maintaining a family-like organizational atmosphere.	①	②	③	④	⑤
7.The shipping organization keeps creating smooth interpersonal relationships among crewmen.	①	②	③	④	⑤
8. The shipping organization places importance on the solidarity of crewmen and their sense of unity.	①	②	③	④	⑤
9. The shipping organization places importance on cooperative relationships and teamwork among crewmen.	①	②	③	④	⑤
10. The shipping organization is highly considerate of crewmen and has interest in them.	①	②	③	④	⑤
11. The shipping organization places importance on performance through competitive climate.	①	②	③	④	⑤
12. The shipping organization places importance on professional knowledge necessary for job performance.	①	②	③	④	⑤
13. The shipping organization gives priority to achieving their goal.	①	②	③	④	⑤
14. The shipping organization has a climate that emphasizes the order of rank.	①	②	③	④	⑤
15. The shipping organization follows formal procedures, regulations and policies.	①	②	③	④	⑤
16. The shipping organization features tight control.	①	②	③	④	⑤
17. The shipping organization follows existing procedures and customs.	①	②	③	④	⑤

Ⅱ. The followings are about organizational support. Please mark your answer with a V shape.

organizational support.	Very Poor	Poor	Fair	Good	Very Good
1. The organization to which I belong puts a high value on my contribution.	①	②	③	④	⑤
2. The organization to which I belong is thankful to me for my extra efforts or overtime work.	①	②	③	④	⑤
3. The organization to which I belong is truly interested in the welfare of crew.	①	②	③	④	⑤
4. The organization to which I belong doesn't disregard the complaints of crewmen at all.	①	②	③	④	⑤
5. The organization to which I belong recognizes that crewmen are doing their best.	①	②	③	④	⑤
6. The organization to which I belong helps crewmen to perform their job by showing what they can do as much as possible.	①	②	③	④	⑤
7. The organization to which I belong values my opinion.	①	②	③	④	⑤
8. The organization to which I belong provides the opportunity of promotion to ensure my growth.	①	②	③	④	⑤
9. The organization to which I belong is willing to help me with interest when I ask something personal.	①	②	③	④	⑤
10. The organization to which I belong is interested in me.	①	②	③	④	⑤

Ⅲ. The followings are about self-efficacy. Please mark your answer with a V shape.

self-efficacy	Very Poor	Poor	Fair	Good	Very Good
1. I believe I can do what's more difficult to do than my current work.	①	②	③	④	⑤
2. I take pride in my ability to work.	①	②	③	④	⑤
3. I like to take care of things in a more advanced way.	①	②	③	④	⑤
4. I am an expert in what I am doing now.	①	②	③	④	⑤
5. I am proud of what I can do.	①	②	③	④	⑤
6. I can do work in an efficient way with other crewmen.	①	②	③	④	⑤
7. When I attempt something, I keep attempting until I carry it out.	①	②	③	④	⑤
8. I believe in myself.	①	②	③	④	⑤
9. When I intend to do something, I start it right away.	①	②	③	④	⑤

Ⅳ. The followings are about perceived fatigue that you felt on board. Please mark your answer with a V shape.

fatigue	Very Poor	Poor	Fair	Good	Very Good
1. I was often in tough situations during job performance.	①	②	③	④	⑤
2. I had difficulty in focusing during job performance.	①	②	③	④	⑤
3. It was difficult for me to watch marine signals.	①	②	③	④	⑤
4. I made frequent technical mistakes while I was working.	①	②	③	④	⑤
5. It's difficult for me to move quickly while I'm working.	①	②	③	④	⑤

Ⅵ. The followings are about the quality of life. Please mark your answer with a V shape.

quality of life	Very Poor	Poor	Fair	Good	Very Good
1. I wasn't nervous on board.	①	②	③	④	⑤
2. I was energetic on board..	①	②	③	④	⑤
3. I wasn't discouraged nor sad on board.	①	②	③	④	⑤
4. I felt confident on board.	①	②	③	④	⑤
5. I had a fun with a light heart on board	①	②	③	④	⑤
6. I wasn't tired nor exhausted on board.	①	②	③	④	⑤

Ⅷ. The followings are about your personal information. Please mark your answer with a V shape..

1. Are you a smoker?

① Yes ② No

2. How old are you?

① In my 20s ② In my 30s

③ In my 40s ④ In my 50s and up

3. What is your educational background?

① With a graduate school education ② With a college education

③ With a junior college education

④ With a high school or lower education

4. What is your employment status?

① Regular worker ② Contract worker ③ Others

5. What is your position?

① Third Officer(engineer) ② Second Officer(engineer)

③ Chief Officer(engineer) ④ Captain(chief engineer)

6. How much are you stressed on board?

① Just a little ② a little

③ a lot ④ quite a lot

7. How long have you served as a crewman?

* Thank you for valuable time.
